# A universal model describing the structure and functions of living systems

**DOI:** 10.1080/19420889.2021.1887549

**Published:** 2021-02-24

**Authors:** Antonis Mistriotis

**Affiliations:** Department of Natural Resources Management & Agricultural Engineering, Agricultural University of Athens, Athens, Greece

**Keywords:** Logic and Life, living systems, self-similarity, maxwell’s demon, information and entropy, thermodynamics

## Abstract

Can Life be explained based on the fundamental Laws of Nature? This question is central in Science since its answer could unify Physics and Biology and open new routes for Medicine. The present study introduces a clear and well-documented hypothesis addressing the unified description of all living systems. The proposed universal model is based on two established characteristics of Life. First, the concept of Functional Self-similarity (FSS) is introduced. As shown by several authors, all living systems can be classified in a multi-level hierarchy of increasing complexity. Systems in all hierarchical levels are characterized by a small set of the same attributes defining Life. This observation implies the existence of an elementary living system (i.e., a quantum of Life) having all the necessary functionalities of living systems. Secondly, the non-equilibrium nature of living systems implies that they should be able to process information since such a function is required for reducing entropy. Therefore, all living systems necessarily perform logical operations similar to electronic circuits. This conclusion, which is based on the requirement to overcome the constraints of the Second Law of Thermodynamics, indicates a close correspondence between living systems and information processing machines, namely computers. Consequently, important theoretical principles and concepts regarding computer design may also apply in the study of living systems. The above considerations lead to the Hypothesis of a Universal Architecture (UAH).

## Life and thermodynamics

The term “living systems” is used to distinguish beings that are alive from abiotic matter. Living systems are characterized by a small set of functional attributes that define Life. These include homeostasis, responsiveness, energy utilization and transformation, growth, evolution, and reproduction [1]. Most of Life’s fundamental characteristics appear incompatible with the simpler abiotic phenomena. For this reason, for a long time, Life was considered as the manifestation of supernatural forces, so it could not be explained in the framework of the Laws of Nature that govern only matter. In particular, growth and evolution are phenomena that increase organization, thus decrease entropy. For this reason, they appear as contradicting the Second Law of Thermodynamics. This discrepancy, however, has been clarified since the monotonous increase of entropy concerns only closed systems in equilibrium, while living systems are far-from-equilibrium open systems [2,3]. Therefore, they can reduce their internal entropy by consuming energy. Nevertheless, the detailed mechanism by which order increases and entropy decreases in living systems has not been satisfactorily elucidated so far.

The present study examines the fundamental physical principles that govern Life. In this context, living systems are studied in terms of their non-equilibrium nature. Following this approach, Life is explained as a far-from-the-equilibrium thermodynamic phenomenon that involves the creation of order (reduction of internal entropy) by accumulating and processing information. The counter-equivalence between information and entropy has been proven in terms of both Mathematics and Physics since 1950 [4,5]. The proposed approach is based on this relationship, which has fundamental implications for the structure and the functionality of living systems.

## Decision-making and entropy

The complexity of structures and forms observed in living systems indicates the versatility of the non-equilibrium processes that are responsible for decreasing entropy. The exploration of the synthetic mechanisms that generate complex structures began early in the 20^th^ century. A good example of these ideas can be found in the work of D’Arcy Thomson [6], who coined the term morphogenesis. His theory assumed that forms are inherent in living systems and their appearance is triggered by specific external factors. Morphogenesis was also studied through mathematical models showing that the emergence of complex structures is possible in open dynamical systems [7,8]. These ideas gained momentum when spontaneous pattern-creation was discovered and mathematically analyzed in physical systems, such as chemical oscillations, e.g., the Belousov–Zhabotinsky reaction [9].

Although these results are impressive and can partially justify the creation of ordered complex structures, they fail to explain the infinite flexibility of living systems that can adapt to variable external conditions by developing new features and “inventing” new skills. Moreover, pattern-creating dynamical models fail to elucidate the qualitative difference between biotic and abiotic systems regarding pattern-formation dynamics. Abiotic dynamical systems may freely undergo transitions from one state of complexity to another when the corresponding control parameters change. For example, a flow can change from laminar to turbulent and back. On the contrary, living systems evolve exhibiting a persistent tendency toward ever-increasing complexity despite temporary failures or setbacks. Mathematical models based on complex dynamics focus mainly on morphogenesis and do not sufficiently address the extraordinary ability of the living systems to adapt and evolve. For this reason, other alternatives are explored in this work.

In 1867, Maxwell proposed a thought experiment, named demon, that, as he believed, was violating the Second Law of Thermodynamics [10]. Maxwell’s demon is a valve separating two chambers, A and B, that contain a quantity of gas in thermal equilibrium. The demon allows the molecules, which have kinetic energy above a threshold, to move toward chamber A, and the molecules with kinetic energy below that threshold toward chamber B. As a result, after a short time, the temperature in chamber A becomes higher than the temperature in chamber B, and the total entropy decreases. In his model, Maxwell neglected the fact that the operation of the valve requires energy consumption and causes total entropy to increase. Therefore, Maxwell’s demon is, in fact, a non-equilibrium open system. Hence, the Second Law is not violated. Despite Maxwell’s error, the demon is a very simple non-equilibrium system where internal entropy decreases similarly to living systems. For this reason, it deserves attention from a new perspective. The mechanism of the demon involves the presence of two states, A and B, and a logical operator, which can classify the gas molecules by their kinetic energy and separate them by processing information. Therefore, Maxwell’s demon demonstrates that a logical processor can decrease entropy.

**Figure 1.**
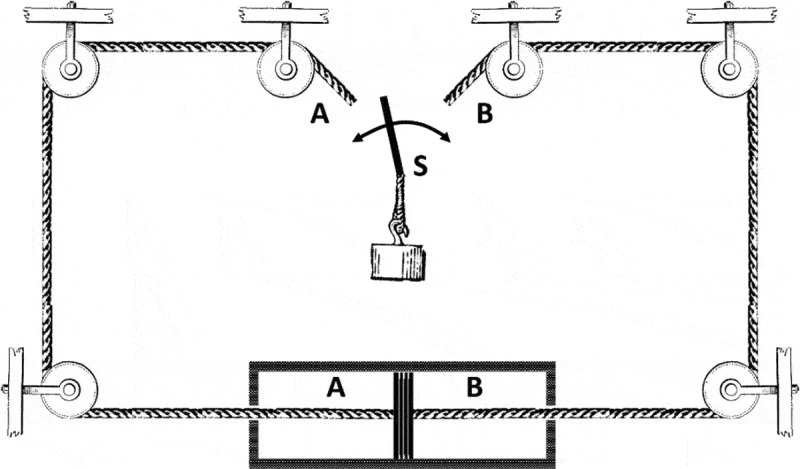
Szilard’s engine: If the particle is in the compartment A, it pushes the piston to the right. Therefore, the switch S must be in position A to move the weight upwards. If the particle is in the compartment B, the switch must be moved to position B so that the weight continues to move in the same upward direction

An alternative version of Maxwell’s demon that directly relates entropy decrease with information processing is Szilard’s engine. Szilard in his effort to resolve the paradox of Maxwell’s demon published a very interesting paper under the title “On the Decrease of Entropy in a Thermodynamic System by the Intervention of Intelligent Beings” [11]. He replaced Maxwell’s demon with a device capable of producing mechanical energy from the kinetic energy of gas molecules in thermal equilibrium. Szilard’s mechanism (Figure 1) consists of a cylinder containing a single gas molecule at a non-zero temperature. A massless piston is inserted at the middle of the cylinder. Therefore, the gas molecule is trapped either in the left or the right half. Then, because of its thermal motion, the particle collides with the piston and moves it to the right if the molecule is trapped in the left half of the cylinder or to the left otherwise. The piston is connected to a weight through a mechanism in such a way that the weight can always move upwards with the help of a switch, S. Specifically, if the switch is in position A and the piston moves to the right, the weight moves upwards, but when the piston moves to the left, the switch must be turned to position B, so that the weight moves again upwards. An intelligent being observes the position of the molecule relative to the piston and operates the switch properly moving the weight always upwards. In this way, if gas molecules are randomly inserted either in the left or the right compartment of the cylinder, the thermal energy of the gas is transformed into mechanical work.

Similar to Maxwell’s demon, information processing by using a logical device (i.e., a switch) is required to make Szilard’s engine function. Later, other studies [4,5] further explored this reciprocal relationship between entropy and information in terms of both Mathematics and Physics. These works showed that thermodynamic entropy and information are physically correlated concepts. Their equivalence is not just formalistic but originates from the probabilistic nature of the thermodynamic phenomena. Macroscopic statistical quantities, such as temperature or entropy, which are used in Statistical Physics and Thermodynamics to describe physical systems, express a loss or lack of information. On the other hand, signal transmission and information processing are physically implemented by electronic devices, while logical values are described by different discrete states of physical systems [12].

**Figure 2.**
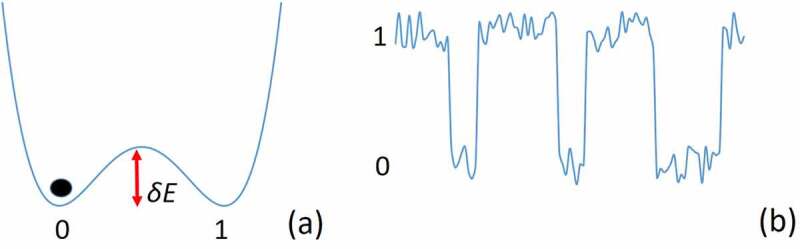
Binary signal produced by an electronic switch with two states

The present discussion shows that a logical device can be used to decrease internal entropy in an open system. However, a switch consumes energy to operate. As Landauer proved, the minimum energy needed by a logic device to function at temperature T is larger than kT, where k is the Boltzmann’s constant [13]. This result, which is known as the Landauer’s Principle, has also been confirmed experimentally [14]. A simplified proof of Landauer’s Principle is the following. A switch is implemented by a physical system at temperature T having two states, 0 and 1, separated by an energy barrier, δE (Figure 2a). The barrier must be larger than the thermal energy of the system to avoid accidental switching that could cause the switch operation to be overwritten by noise (Figure 2b). Therefore, δE>kT. When the device switches from, e.g., state 0 to state 1, energy larger than δE must be given to the system to cross the barrier (Figure 2a). After the system changes state, its energy must be dissipated to avoid accidental bouncing back to state 0. Therefore, cooling down to temperature T is necessary, so energy in the form of heat is released to the environment.

In conclusion, Maxwell’s demon or Szilard’s engine are non-equilibrium devices, which can reduce the internal entropy of a physical system by processing information. In this context, these thought experiments are interesting, simplified models that simulate the mechanism by which internal entropy decreases in living systems. Therefore, logic constitutes an indispensable attribute of Life that is manifested as responsiveness and decision-making. Such functions are apparent not only in advanced living systems, for example, animals, but they are also present in simpler forms of Life, including unicellular organisms [15–17], as will be explained below.

## Functional self-similarity and the elementary living system

Several studies [1,18] have shown that biological systems form a hierarchy of increasing complexity. The term complexity describes synergic collective behavior so that a system cannot be merely considered as the superposition of its parts. Living systems are complex in this sense. In the living systems hierarchy, advanced forms consist of components that are also characterized as living and interact with each other. For example, the human body contains organs, multicellular organisms are made of millions of cells, and cells enclose organelles. In this way, complexity increases with the hierarchical levels since systems in each scale are composed of constituent parts that are also complex but of lower complexity. In other words, higher complexity levels enclose the complexity of the lower scales.

All systems belonging to the hierarchy of Life are alive since they satisfy all the required basic characteristics, such as growth, reproduction, or reaction to external signals with the same competence, whether they are mammals or just cells. Therefore, the attributes of Life do not change qualitatively as complexity increases or decreases. In other words, all living systems can perform the same basic functions independently of their complexity level. Their position in the hierarchy merely determines the degree of sophistication regarding the expression of these fundamental functional properties. There is no limit in moving upwards in the living systems hierarchy toward ever-growing complexity. On the other hand, if the descending direction is followed, reducing complexity results in abiotic matter. Therefore, there must be an elementary system of minimum complexity, which satisfies the definition of Life. Following this approach, we may assume that an elementary living system exists that exhibits all the basic attributes defining Life.

The above-described hierarchy of increasing complexity that classifies living systems reminds fractals. Fractals are mathematical models with an impressively complex geometrical structure generated by repeating a simple elementary transformation [19,20]. In other words, an elementary pattern with fixed geometric characteristics is recursively applied to produce the fractal geometry, so that the basic geometric properties remain unchanged as the complexity of the fractal grows. For this reason, fractals are characterized as self-similar structures. The properties preserved throughout the living systems hierarchy are functions, such as metabolism, growth, reproduction, and responsiveness. As the systems complexity increases, these functions are implemented through more sophisticated mechanisms, but the functionalities remain the same. Therefore, another type of self-similarity can be defined based on the preservation of functions in multiple scales. We call this Functional Self-similarity. As fractal geometry requires an elementary pattern that acts as a seed, Functional Self-similarity implies the existence of an elementary living system [21].

As explained in the previous section, Maxwell’s demon is a simple, attractive model simulating an elementary system that decreases entropy. The central element of this model device is a logic switch, which can separate the particles of a single macrostate in two others by increasing the information of the system. This operation, which, consequently, decreases the system’s internal entropy, is only possible at the cost of energy, as Landauer’s principle explains. On the other hand, the newly generated information may be exploited for enhancing energy acquisition from the environment. A good example of this process is Szilard’s engine (Figure 1), which can produce mechanical work from the thermal energy of a system in equilibrium with the help of its intelligent switch.

**Figure 3.**
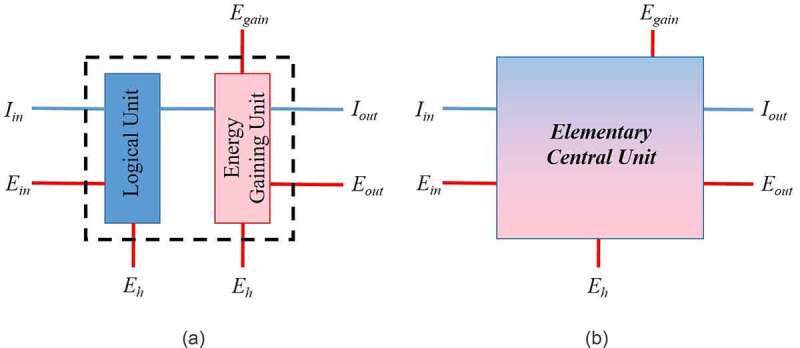
The central unit of an elementary living system: a) explicit version, b) simplified version

Therefore, an elementary device can be theoretically constructed, which models the above-described two-stage operation (Figure 3a). It consists of two units performing two distinct tasks. First, a Logical Unit (LU), namely a switch, consumes energy, and processes the incoming information. In the second component of the device, called the Energy Gaining Unit (EGU), information is used to gain energy from the environment.

This model can be further simplified by ignoring its internal structure, so it takes the form of Figure 3b. The device shown in Figure 3b amplifies information, so I_out_ is larger than I_in_. On the other hand, energy is preserved according to the First Law of Thermodynamics. Therefore,

$$E_{in}+\;E_{out}+\;E_{gain}+\;E_{h}=\;0$$

assuming that the incoming energy, E_in_ and E_gain_, is positive and the outgoing, E_out_ and E_h_, negative. The form of energies E_in_ and E_out_ must be suitable to support the operation of the Logical Unit (e.g., chemical or electrical). On the contrary, energy E_gain_ is of any arbitrary form (e.g., thermal, or chemical but possibly incompatible to the Logical Unit), so the amplified information I_out_ is necessary to capture it from the environment and transform it to a “useful” form (i.e., E_out_). A part of both incoming energies, E_gain_ and E_in_, is lost as heat, E_h_, in agreement with Landauer’s principle. If |E_out_| is larger than E_in_ the system is sustainable. Otherwise, it decays. In the case that |E_out_| > E_in_, a part of E_out_ can be fed back as input, E_in_, to the Logical Unit (LU), so the system becomes self-powered. In this case, the model resembles an order-creating, autocatalytic chemical system. Specifically, a part of the energy output, E_out_, feeds back the Logical Unit, which in turn supplies the Energy Gaining Unit with increased information, I_out_. In this way, the two sub-units develop a cross-catalytic relationship, which is typical in order-creating chemical models [9]. The model unit, shown in Figure 3b, is named Elementary Central Unit (ECU).

It is important to note that there is an amplifying catalytic relation between the information I_out_, which activates the EGU, and the gained useful energy, E_out_. The ability of the EGU to transform E_gain_ into useful energy, E_out_, depends on the amount of information I_out_, which is provided by the LU to the EGU as input. Intuitively, this mechanism is justified by various examples. Knowledge in human societies has been historically linked to the development of skills, such as agriculture and animal husbandry that made larger quantities of food of higher nutritious value available. Similarly, in animal groups, the development of feeding or hunting strategies is related to the animals’ welfare.

The amplification of the EGU’s efficiency in gaining energy due to information can also be shown mathematically by considering a two-bit Szilard’s engine. A Szilard’s engine, which can store and process more information, gains more energy than the standard model presented in Figure 1. In Szilard’s single-particle model, a logical process implemented by a switch can transform the random motion of a piston into the systematic upward motion of a weight. The proper switch’s operation requires the detection of the particle’s position so that the random thermal motion of the particle can be used to move the weight to the correct upward direction. In Szilard’s model, the position of the particle is determined by a single bit. Therefore, the piston is always placed in the middle of the cylinder. In a two-bit Szilard’s engine, the position of the particle can be determined more accurately, since four different states can be distinguished. Specifically, if L is the length of the cylinder containing the particle, the four states are defined as:

1)0≤x<L/4;2)L/4≤x<L/2;3)L/2≤x<3L/4;4)3L/4≤x<L

Where x is the position of the particle. If the particle is in the states 2 or 3, the procedure of the one-bit engine is applied, so no additional work is gained. However, if the particle is in the states 1 or 4, the massless piston can be inserted at the positions L/4 or 3L/4, respectively. In such cases, the weight is displaced over a longer distance, equal to 3L/4, so the produced work increases by 50%. Therefore, increased information results in an EGU of higher efficiency.

Elementary units can be linked together to form an integrated system. In this way, sustainable living systems can be created by interconnecting many ECU’s. In this case, the output energy, E_out_, or the output information, I_out_, of one unit can be the input, E_in_ or I_in_, of another. Such a network is expected to increase its total outgoing energy, and in parallel, to amplify its information output, causing its internal complexity to increase. The dual nature of the Elementary Central Unit (ECU), as shown in Figure 3, requires the transfer of both energy and information to retain the functional connectivity of an integrated network system built of many interlinked ECUs. Consequently, dual connecting pathways are necessary. As a result, two parallel networks must develop. This conclusion reminds the two parallel networks of nerves and veins in animals’ bodies. In specific cases, for reasons of convenience, the two networks may also use the same pathways. For example, chemical signals, secreted in the form of hormones, are transmitted through the cardiovascular system of the body, which is also used for transferring energy in the form of sugars. The same dual network can also be observed in human societies, where functional units, such as cities or villages, are connected by transportation pathways, namely roads, railroads, etc., and by communication lines, namely telephone lines.

## Life’s Universal Architecture

As shown in the previous section, any living system necessarily contains a logic device as a means to overcome the constraints of the Second Law of Thermodynamics. Moreover, it was explained that living systems exhibit the same functionalities independently of their complexity so that Functional Self-similarity can be established throughout Life’s hierarchy. Such common functionalities imply the general validity of a set of functional and structural principles that can be described as a universal model. This concept of a universal model reminds the standardized methodology, called von Neumann architecture [22], which is the basis for the design of computers. Considering the importance of logical operations in living systems, this similarity between biological organisms and computers may not be just a coincidence. The general structure of a computer, according to von Neumann, consists of:

1. The Control Unit which reads and interprets the instructions stored in the memory and executes them.
2. The Memory where both instructions and data are stored.
3. The Arithmetic and Logical Unit, that is a special-purpose processor where all binary operations are performed.
4. Input and output (I/O) units operated by the Control Unit.

The above structure of computers is almost universal and applies to today’s general-purpose machines independently of their scale [23]. By adopting the term used for computer design, we call architecture the set of the fundamental principles governing the structure and the functionalities of a living system. These considerations lead to the Universal Architecture Hypothesis (UAH), claiming that all biological systems have the same general structure in close analogy with computers [21]. In the present section, we attempt to analyze the basic features of the Living System Architecture (LSA).

**Figure 4.**
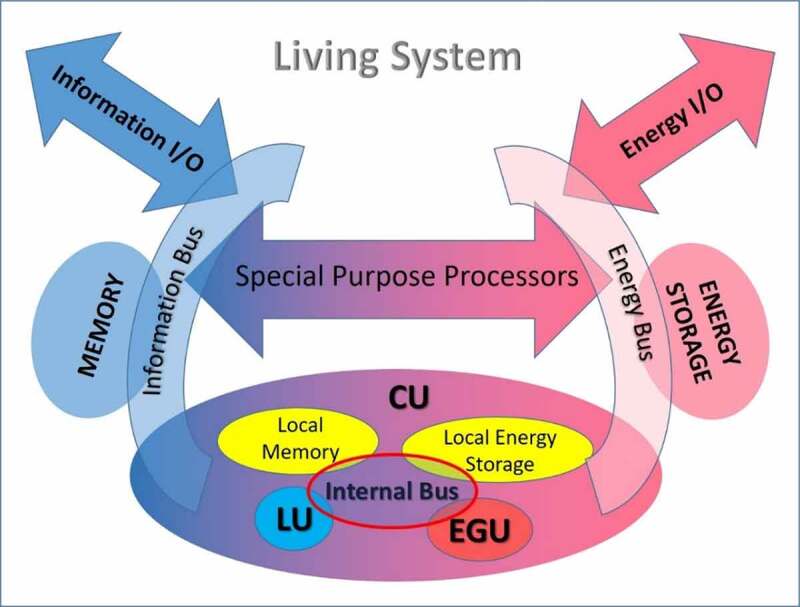
The basic structure of a living system

The computer design principles, according to von Neumann’s architecture, may be used as a guide for understanding Living System Architecture since a logic system is a necessary component of any living system (Figure 3). Therefore, a control unit, memory units, input-output channels, and specialized logical processors dedicated to specific frequent operations should also be included in LSA. A signal transferring pathway, the information bus, connects all components (Figure 4). Besides their information-handling sub-system that exists in computers, the biological organisms also process energy. Consequently, living systems must also be equipped with additional energy-related units, so their structure supersedes that of a logic device. The cross-amplifying relation between the two sub-units of the ECU (Figure 3) is a key feature of living systems and distinguishes them from simple logic devices, such as computers.

Following the above arguments, LSA (Figure 4) is structured around a Central Unit (CU). The Central Unit (CU) consists of a network of ECUs (Figure 3), just as the Control Unit of a computer is made of gates. As a result, CU exhibits the same functional features as the ECU. Specifically, the Central Unit, to accomplish its tasks, consists of two complementary sub-units: a) a logic device for processing information, and b) an energy gaining organ that can capture useful energy from the environment. The logic device processes information by consuming energy. The energy gaining unit exploits the stored information to assimilate energy from the environment. The two sub-units have a cross-amplification relationship, which is the key element of an Elementary Living System, as discussed in the previous section, and characterizes all living systems.

In von Neumann Architecture, memory is a necessary important component of all logic systems. Complex logic operations cannot be performed without a large enough information storage unit [22]. For this reason, memory units are built in all logic circuits to support their operation. In chips, memory units are inserted in the design at a close distance from the corresponding gates. Special purpose storage units, such as cache memory or registers [23], which are built in the Central Processing Unit (CPU), greatly facilitate operations. In the same way, living systems are equipped with memory units that support information processing. As in logic devices, fast local memory units in proximity to logical units that store information for immediate use by the corresponding processor are common in living systems. Local memory is a key element of LSA due to the chemical character of biological processes. When reacting chemicals are transported by diffusion, which is a very slow physical process, reactants have to be kept at a short distance from the processing unit. Besides the local information-storage units, living systems also have local units storing energy. In this way, the energy needs of the processor are promptly satisfied. In the energy processing sub-unit, energy storage units exist, which can feed the Central Unit on demand, acting equivalently to memory.

The information bus is a device that provides communication between all units of the logic processor (Figure 4). In computers, the bus is implemented as a data transferring pathway that connects the Control Unit with the memory and the Arithmetic and Logical Unit. A similar information-carrying channel is a necessary component of living systems logic sub-unit. Moreover, living systems process energy. Therefore, an energy-transferring channel, which we name energy bus, running in parallel to the information bus, must be added to the structure (Figure 4). The energy bus transports assimilable, order-creating energy quantities, such as E_in_ and E_out_ (Figure 3). The cross-amplifying relationship between these two sub-unit types requires a dual communication network connecting the components of a complex living system. Malfunctions in any of these two complementary networks may cause severe failures to the system since, in this case, the cross-amplification interaction between the two sub-units breaks down.

Similar to logic devices, Living Systems may also include special-purpose processors. In organisms, specialized subsystems are responsible for performing frequent, high-priority tasks. For example, in cells, mitochondria are organelles in charge of the cell’s metabolic activities [24]. Likewise, other organelles have different targeted missions to accomplish. In multicellular organisms, organs play the role of specialized processors, such as the heart that is a processor responsible for maintaining the flow of energy and information in the system’s central bus, namely the cardiovascular system. Special-purpose processors have a similar internal structure to the Central Unit. They include both a Logical and an Energy Gaining Unit and communicate through an internal bus with local memory units and with the Central Unit through the central bus.

Input/output communication channels are necessary components in computer design. They transfer data to a display or an input device, or any other interface linking the system with the outside world. The communication channels may also connect the device with other systems through a communication network. Similar organs that transfer information to or from a living system are also required in LSA. Moreover, energy-transferring input/output channels must also exist. Energy input organs provide the E_gain_ energy to the Central Unit while a “cooling” device (energy output) removes waste energy, E_h_, and residues.

The above schematic description of the Living System Architecture (LSA) was based only on theoretical considerations. The proposed structure is necessary to overcome the constraints of the Second Law of Thermodynamics and decrease the internal entropy of a living system. Since a logic organ is a necessary component of a living system, LSA includes a logic sub-unit that is structured following the basic computer design principles. A similar structure is proposed for the energy-processing sub-unit that pairs the logic sub-unit. Although the implementation of the LSA may differ between systems of the same or different scales, the basic structural and functional characteristics remain the same in agreement with the Functional Self-similarity principle. In the following section, an example will be analyzed to demonstrate the validity of the Universal Architecture Hypothesis.

## A case study – Eukaryotic cells

The Universal Architecture Hypothesis is proposed as a unification scheme that applies to all hierarchical levels of Life, including cells, multi-cellular organisms, and societies. In some scales of the living system hierarchy, the LSA structure is rather obvious. For example, in mammals’ bodies, the information and energy processing sub-units can be easily recognized. On the other hand, the relevance of the UAH to cells is more difficult to show. In particular, decision-making has been believed for centuries as a functionality of advanced organisms only, so it was considered missing in simple living systems such as the cells. Nevertheless, decision-making is a key feature of the information-processing unit, thus LSA cannot function without it.

In this section, we will investigate chemical processes that take place at cellular level and involve decision-making implemented by chemical switches. Allosteric regulation of enzymes is such an example case [25]. Enzymes are proteins that act as catalysts in various biochemical reactions by connecting to other molecules called ligands. Allosteric inhibition occurs when regulatory molecules, called effectors, bind to the enzyme and modify its structure, so it dissipates the enzyme’s catalytic efficiency. Biochemical control is actually an analog process rather than a digital one. Nevertheless, enzymatic regulation appears similar to a digital (binary) ON/OFF switch since enzymes are very efficient catalysts that can accelerate the process by orders of magnitude. In this way, allosteric control resembles switching between two distinct states.

Another complex biochemical function resembling the execution of an instruction stored in memory is the synthesis of proteins through translation [26]. Translation is preceded by the transcription process by which an mRNA molecule is produced from the corresponding DNA sequence. Transcription is usually triggered by an external signal reaching the cell through its plasma membrane. The incoming signal initiates a process fetching the data encoding the protein structure from the DNA memory. Information is transferred with the help of an mRNA molecule, which acts as a data carrier. The signal (mRNA) propagates through the cytoplasm to the ribosomes, which are processors able to translate (decode) it into protein molecules. The produced proteins are released either in the cytoplasm for internal use or secreted through the plasma membrane to the external environment. The cell’s components that participate in the synthesis of proteins can be also recognized in the UAH model of Figure 4. Specifically, DNA corresponds to the central memory of the system. The mRNA molecules can be considered as equivalent to the local memory. The ribosomes act as special-purpose processors for the translation. Finally, the cytoplasm allows the communication between these components, so it corresponds to the bus, while the plasma membrane is the main input-output channel for both information and energy.

What is clearly missing in the above scheme is a Control Unit, namely an organ that interprets extracellular or intracellular signals and issues the corresponding instructions to the DNA, thus initiating transcription. Such an organ that makes decisions on gene expression could be either centralized or distributed. Gene expression is controlled by a biochemical mechanism called regulated transcription [27]. Regulated gene expression appears most impressively during the developmental phase of a multicellular organism, when cells of the same genetic origin execute different instructions, produce different proteins, and develop into morphologically distinct types. As a result, cells differentiate and form tissues and organs.

Gene expression in eukaryotic cells is controlled by specialized proteins called transcription factors (TF) [27]. Each gene is activated by a combination of several co-regulating TFs that form a complex. TF co-regulating complexes act as decision-making and control organs. TFs of eukaryotic cells regulate transcription by controlling the binding of a protein catalyzing mRNA synthesis, called RNA-polymerase, to a specific site of DNA, called promoter. The regulatory function of TFs is implemented by many interrelated processes. TF complexes contain a DNA-binding domain, called activator, which attaches to a transcription-control element of the DNA, named enhancer. Additionally, certain TFs in the co-regulating complex contain signal-sensing domains, so they can detect intercellular signals by connecting to ligands. The TFs which are equipped with a sensing domain can transmit the signals to the rest of the co-regulating complex. Moreover, the regulation mechanism involves several components with complementary action that can interact with the DNA and reshape its three-dimensional structure. The DNA of eukaryotic cells is large, so it is condensed when being inactive. In condensed form, it does not allow RNA-replicase to reach the promoters and initiate transcription. For this reason, activators first turn chromatin into an “open” state. The above-described processes are coordinated and form an integrated control mechanism. The distance between the regulating elements (enhancer) and the promoter facilitates the operation of large regulating organs, namely TF complexes [27]. The concentrations of the various TFs in the nucleus control the expression of different genes in a specific cell. Nuclear TFs concentrations are regulated by extracellular signals. Special transmembrane receptor proteins transduce external signals to the inner cell and finally to the TFs in the nucleus. Various ligands can pass through the plasma and the nucleus membranes and bind to the corresponding TFs. The so activated TFs bind to the DNA regulatory element of the relevant gene [27].

Recent works [28] present evidence showing that the regulatory proteins of the cell, namely the TFs, form an integrated system, which can be viewed as a complex network of multiply interacting components. For example, the human genome contains more than 2000 co-regulating TFs. This network is internally structured in hierarchical levels of various degrees of connectivity. Graphical analysis of the regulatory interactions between TFs indicates the presence of modular structures and hubs, which implies the existence of global regulators in the transcription factors network. Therefore, the cell’s regulatory system should be viewed as an integrated, highly structured organ. This regulatory organ of the cell coexists with its memory, namely the DNA molecules, in the nucleus. The nuclear membrane encloses both systems that are critical for the functionality of the cell, so it protects them and increases the efficiency of their interaction by compartmentation.

In conclusion, cells are equipped with a central logic unit in the form of a sophisticated regulatory system, which can process signals and make decisions in line with the UAH. The logical processes performed by the cell’s logic unit closely resemble a computer instruction cycle. First, an input signal in the form of a ligand reaches the processor. Then, an instruction is issued as an activated TF. The co-regulating TFs bind to specific enhancers in DNA, which is the cell’s main memory, and initiate the synthesis of the corresponding mRNA molecules. Finally, the message carried by the produced mRNA is decoded at the ribosomes to produce an executable command-protein. Recent studies on the cognitive abilities of unicellular organisms [15,17] have provided evidence that confirms the presence of decision-making functions in such organisms, thus further supporting the above conclusion.

We remind that the Universal Architecture scheme, proposed in the previous section, has not been derived by induction from experimental data but is imposed as a necessity to overcome the constraints of the Second Law of Thermodynamics in a non-equilibrium complex physical system. The evidence discussed in this section simply confirms UAH’s validity.

## Conclusions

The present study presents a model, named Universal Architecture Hypothesis (UAH), that describes the principles governing the structure and the functions of living systems. UAH assumes the following structure for all living systems:

1)A Logic Unit is used to increase the information contents of the system by consuming energy. Therefore, it works as Maxwell’s demon, reducing the internal entropy of the system. Self-organization, self-regulation, response to external signals, adaptive behavior, and evolution are functions emanating from the Logical Unit.

2)An Energy Gaining Unit exploits the stored information to gain energy from the environment and transforms it into assimilable energy, which can be used by the Logical Unit of the system itself or that of other connected systems. The Energy Gaining Unit is responsible for metabolism and growth.

3)A Memory Unit is necessary for storing information. Any advanced operation involving information processing requires a large enough memory. The stability of living systems’ memory is a critical constraint that adds important required functionalities to the system. Specifically, defense and reproduction functions aim to enhance memory robustness.

4)A dual communication network connects the units of the system, allowing the transfer of both information and energy. Good coordination of energy and information transfer is necessary to avoid disharmony, which may cause stress and severe failures in the network. Strong synergic actions lead to the synthesis of advanced integrated systems of higher complexity.

Therefore, the basic attributes of Life are inherently described in the living systems’ Universal Architecture. It is not easy to recognize all elements of the Living System Architecture in all scales of Life’s hierarchy. However, as discussed above, recent discoveries confirm its validity not only in advanced forms of Life, such as mammals, but also at the cellular level [15–17], or in plants [29]. Moreover, the development of biological (DNA) computers [30] is another good example that demonstrates the connection between logical and biochemical processes, thus confirming the relevance of logic even in low-level biological systems.

## References

[1] Raven PH, Johnson GB, Mason KA, et al. Biology. 11th edition. New York: McGraw-Hill Education; 2017. Chapter 1.

[2] Schrödinger E. What is life? Cambridge: Cambridge University Press; 1944..

[3] Prigogine I. From being to becoming. New York: W.H. Freeman and Company; 1980.

[4] Shannon CE. A mathematical theory of communication. Bell Syst Tech J. 1948;27(3):379–423.

[5] Brillouin L. The negentropy principle of information. J. of Applied Physics. 1953;24(9):1152–1163.

[6] Thomson DW. On growth and form. Cambridge: Cambridge University Press; 2nd edition; 1945. First published in 1917.

[7] Turing AM. The chemical basis of morphogenesis. Philosophical Transactions of the Royal Society of London. Series B, Biol Sci. 1952;237:37–72.

[8] Thom R. Structural stability and morphogenesis. Reading, Massachusetts: W.A. Benjamin Inc; 1975. English translation by DH Fowler.

[9] Nicolis G, Prigogine I. Self-organization in non-equilibrium systems. New York: John Wiley & Sons; 1977.

[10] Thomson W. Kinetic theory of the dissipation of energy. Nature. 1874;9(232):441–444.

[11] Szilard L. On the decrease of entropy in a thermodynamic system by the intervention of intelligent beings. In: Leff HS, Rex AF. editors. Maxwell’s demon: entropy, information, computing. Princeton, New Jersey: Princeton University Press; 1990.page 124–133.

[12] Maruyama K, Nori F, Vedral V. Colloquium: the physics of Maxwell’s demon and information. Rev Mod Phys. 2009;81(1):1–24.

[13] Landauer R. Irreversibility and heat generation in the computing process. IBM Journal of Research and Development. 1961 7;5(3):183–191.

[14] Berut A, Arakelyan A, Petrosyan A, et al. Experimental verification of Landauer’s principle linking information and thermodynamics. Nature. 2012;483(7388):187–189. 22398556 10.1038/nature10872

[15] Nakagaki T, Yamada H, Tóth Á. Maze-solving by an amoeboid organism. Nature. 2000;407(6803):470. 11028990 10.1038/35035159

[16] Baluška F, Mancuso S. Deep evolutionary origins of neurobiology – Turning the essence of ‘neural’ upside-down. Commun Integr Biol. 2009;2(1):60–65. 19513267 10.4161/cib.2.1.7620 PMC2649305

[17] Vallverdú J, Castro O, Mayne R, et al. Slime mould: the fundamental mechanisms of biological cognition. BioSystems. 2018;165:57–70. 29326068 10.1016/j.biosystems.2017.12.011

[18] Miller JG. Living systems. New York: McGraw-Hill Inc; 1978.

[19] Feder J, Fractals. New York: Springer Science+Business Media; 1988.

[20] Takayasu H. Fractals in the physical sciences. Manchester: Manchester University Press; 1990.

[21] Mistriotis A. Self-similarity in living systems. Amazon.com Inc.; 2018 [accessed on December 15, 2020]. Kindle e-book: https://www.amazon.com/Self-similarity-Living-Systems-Antonis-Mistriotis-ebook/dp/B07G2DVMDL

[22] Von Neumann J. First draft of a report on the EDVAC. Moore School of Electrical Engineering, University of Pennsylvania; 1945.

[23] Patterson DA, Hennessy JL. Computer organization and design. 5th edition. Oxford: Morgan Kaufmann; Elsevier Inc; 2014.

[24] Raven PH, Johnson GB, Mason KA, et al. Biology. 11th edition. New York: McGraw-Hill Education; 2017. Chapter 4.

[25] Lodish H, Berk A, Kaiser CA, et al. Molecular cell biology. 7th edition. New York: W. H. Freeman and Co.; 2013. Chapter 3 - Protein Structure and Function.

[26] Lodish H, Berk A, Kaiser CA, et al. Molecular cell biology. 7th edition. New York: W. H. Freeman and Co.; 2013. Chapter 4 - Basic Molecular Genetic Mechanisms.

[27] Lodish H, Berk A, Kaiser CA, et al. Molecular cell biology. 7th edition. New York: W. H. Freeman and Co.; 2013. Chapter 7 - Transcriptional Control of Gene Expression.

[28] Babu MM, Luscombe NM, Aravind L, et al. Teichmann SA. Structure and evolution of transcriptional regulatory networks. Curr Opin Struct Biol. 2004;14(3):283–291. 15193307 10.1016/j.sbi.2004.05.004

[29] Brenner ED, Stahlberg R, Mancuso S, et al. Plant neurobiology: an integrated view of plant signaling. Trends Plant Sci. 2006;11(8):413–419. 16843034 10.1016/j.tplants.2006.06.009

[30] Adelman LM. Molecular computation of solutions to combinatorial problems. Science. 1994;266(5187):1021–1024. 7973651 10.1126/science.7973651

